# Effect of Hexagonal Boron Nitride Nanoparticles on the Push-Out Bond Strength of a Bioceramic Root Canal Sealer

**DOI:** 10.3390/nano16211447

**Published:** 2026-11-08

**Authors:** Ismail Ozkocak, Ikbal Sena Celebi Keskin, Banu Arıcıoglu

**Affiliations:** Department of Endodontics, Faculty of Dentistry, University of Istanbul Medeniyet, Istanbul 34956, Türkiye

**Keywords:** bioceramic sealer, nanoparticles, hexagonal boron nitride, bond strength

## Abstract

This in vitro study evaluated the effect of hexagonal boron nitride (HBN) nanoparticles on the push-out bond strength of a bioceramic root canal sealer. Sixty-eight extracted human maxillary central incisors were prepared using Reciproc R40 instruments and assigned to four groups: Adseal, unmodified BioRoot RCS, and BioRoot RCS containing 5% or 20% HBN by volume. Following obturation with gutta-percha and the respective sealers, specimens were stored at 37 °C and 100% humidity for 14 days. One-millimeter-thick slices from the coronal, middle, and apical thirds underwent push-out testing. Data were analyzed using a linear mixed-effects model, with sealer type, root region, and their interaction as fixed effects and tooth identity as a random effect (*p* < 0.05). BioRoot RCS containing 20% HBN showed significantly higher bond strength than Adseal in the coronal region, no significant difference in the middle region, and significantly lower bond strength in the apical region. Unmodified BioRoot RCS showed the lowest bond strength in all regions. Regional variations differed according to sealer type. Within the limitations of this study, incorporating 20% HBN increased the push-out bond strength of BioRoot RCS. Further investigation of the physicochemical, biological, and long-term properties of HBN-modified sealers is required.

## 1. Introduction

The success of endodontic treatment depends in part on the quality of root canal obturation, which aims to prevent microleakage and bacterial infiltration [1]. To achieve an effective seal, root canal sealers play a crucial role in the obturation process by filling voids between the canal walls and core materials [1,2]. Over the years, various types of root canal sealers, such as resin-based sealers (RBSs) and bioceramic sealers (BCSs), have been developed and evaluated for their bond strength, biocompatibility, and sealing ability [2,3,4]. Although RBSs are widely used because of their favorable physicochemical properties, including strong adhesion to dentin, dimensional stability, and good handling characteristics, concerns regarding their limited bioactivity and biological performance have encouraged the development of alternative root canal sealers [4,5,6]. BCSs have gained popularity due to their biocompatibility, bioactive characteristics, ability to promote apatite formation, and potential role in bone healing [7,8]. These sealers are characterized by their alkaline pH, calcium ion release, and favorable sealing-related properties [2,9]. Despite these advantages, some BCSs also have disadvantages, such as taking longer to set and having higher solubility [8]. Therefore, different additives have been incorporated into BCSs to improve their physicochemical properties, including setting characteristics [10].

Nanoparticles have been increasingly investigated in dental materials because of their potential to enhance mechanical performance and physicochemical properties [10,11,12]. Hexagonal boron nitride (HBN) nanoparticles have attracted considerable attention in biomedical applications due to their high mechanical strength, thermal stability, chemical resistance, and favorable biocompatibility [13,14,15]. HBN nanoparticles have previously been used as reinforcing agents in dental materials such as glass ionomer cements, resulting in improved mechanical properties [16]. Furthermore, Baghdadi et al. investigated the incorporation of boron nitride (BN), titanium carbide (TC), and multi-walled carbon nanotubes (MWCNTs) into BioRoot RCS and evaluated several physicochemical properties, including setting time, solubility, elution, and pH [10]. However, despite these promising findings, the potential effects of HBN incorporation into BCSs remain insufficiently investigated. In particular, the influence of different HBN concentrations on push-out bond strength in the coronal, middle, and apical regions of the root canal has not yet been investigated.

Therefore, the aim of the present study was to evaluate the effect of incorporating different concentrations of HBN nanoparticles into a BCS on its push-out bond strength and to compare the results with those obtained using a conventional RBS and an unmodified BCS. The null hypothesis was that the incorporation of 5% and 20% HBN would not significantly affect the bond strength of the BCS.

## 2. Materials and Methods

Ethical approval was obtained from the Ethics Committee of the Faculty of Dentistry at Istanbul Medipol University (approval no. 993).

A sample size calculation was performed using G*Power 3.1 for Windows (Heinrich Heine University, Düsseldorf, Germany) based on a repeated-measures MANOVA model for between-group effects. The calculation assumed a medium effect size (Cohen’s f = 0.25), a significance level of α = 0.05, a statistical power of 80%, four experimental groups, and three repeated measurements corresponding to the coronal, middle, and apical root regions. The minimum required sample size was determined to be 64 teeth (16 teeth per group). Sensitivity analyses using assumed correlations of 0, 0.3, and 0.5 among repeated measurements yielded the same minimum sample size. To account for potential specimen loss or experimental failures during preparation and testing, the sample size was increased to 17 teeth per group. Accordingly, a total of 68 human maxillary central incisors, recently extracted for periodontal reasons, were included in the study.

Teeth with similar root diameter and round canal morphology, without cracks or fractures, were included in the study. Root canals were checked with periapical radiography. The crowns of the teeth were sectioned at the cemento-enamel junction using a steel separating disc under continuous water cooling. Working length was determined by advancing a 10 K-type file (Dentsply, Maillefer, Ballaigues, Switzerland) into the canals until it was visible through the apical foramen and then withdrawing 1 mm from this length. The glide path was made manually with 10, 15 and 20 K-files. All root canals were instrumented up to size Reciproc #40 (VDW, Munich, Germany). The irrigation was performed with a 30-G side-vented needle using 2 mL of 5.25% sodium hypochlorite (NaOCl) between used files (#25 and #40). The final irrigation protocol consisted of 2 mL of 5.25% NaOCl, followed by 2 mL of saline, 2 mL of 17% EDTA for 1 min, a further rinse with 5.25% NaOCl for 1 min, and a final saline rinse. The root canals were dried with size 40/0.06 paper points.

Root canals were obturated with:

Group 1: (Control group): Gutta-percha and RBS (Adseal, Meta Biomed, Cheongju, Republic of Korea).
Group 2: Gutta-percha and BCS (BioRoot RCS, Septodont, Saint Marcellin, France).
Group 3: Gutta-percha and BCS containing 5% by volume of HBN nanoparticles (Molchem, Nanokar Nanotechnology, Istanbul, Türkiye).
Group 4: Gutta-percha and BCS containing 20% by volume of HBN nanoparticles.

The chemical compositions and manufacturers of the root canal sealers evaluated in the present study are summarized in Table 1.

The HBN incorporated into the BCS was supplied as powder particles with a size range of 40–50 nm (Molchem, Nanokar Nanotechnology, Istanbul, Türkiye). To prepare the modified sealers, the BCS powder and HBN particles were first measured volumetrically using graduated tubes. The powders were then transferred to a separate container and manually mixed. Subsequently, the mixture was placed in a capsule mixer and shaken for 15 s to obtain a homogeneous distribution of HBN particles within the sealer powder before the addition of the liquid component. Following homogenization, the modified powder was mixed with the manufacturer’s recommended liquid ratio without adding any additional liquid.

Specimens were stored in an incubator at 37 °C and 100% humidity for 2 weeks. The 14-day storage period was selected to provide a standardized period for the initial setting and maturation of the tested sealers before push-out testing, consistent with previous studies investigating calcium silicate-based endodontic materials [17,18].

Dentin discs with a thickness of 1 mm were obtained from the coronal, middle and apical regions of roots. After marking the roots, sections were taken at distances of 3 mm, 8 mm, and 13 mm from the apex of the root. To consider the same level of the canal, teeth were chosen with the same length and embedded in resin. The thickness of the specimen was measured using a digital caliper with an accuracy of 0.001 mm. Before testing, each specimen was positioned in the universal testing machine and the plunger tip was carefully aligned with the sealer surface. Using a dental loupe under ×5 magnification (EyeMag Pro; Carl Zeiss, Oberkochen, Germany) and an external light source, it was confirmed that the plunger contacted only the sealer material without touching the surrounding dentin walls or gutta-percha. The specimens were placed in an Instron test machine and a force with apico-coronal direction was applied at a crosshead speed of 1 mm/min. The maximum push-out force was recorded and the bond strength (MPa) was calculated by dividing force by the area of the bonded interface. The lateral surface areas of the truncated cone were calculated as 2.77 mm^2^, 2.2 mm^2^, and 1.51 mm^2^ from the coronal to the apical regions, respectively.

For SEM evaluation, representative specimens prepared from the extracted human teeth included in the study were treated with 37% orthophosphoric acid for 1 min to remove the surface smear layer. Following rinsing with distilled water, the specimens were dehydrated with alcohol and allowed to dry before examination using a scanning electron microscope (Thermo Scientific Quattro S, Thermo Fisher Scientific, Waltham, MA, USA) at ×2000 and ×5000 magnifications. The SEM analysis was performed to evaluate the surface morphology of the sealers and the distribution of HBN particles within the BCS matrix.

Statistical analyses were performed using SPSS software (version 25.0; IBM Corp., Armonk, NY, USA). Push-out bond strength values were calculated in megapascals (MPa) by dividing the maximum dislodgement force (N) by the bonded interface area (mm^2^). Descriptive data were expressed as means and standard deviations (SD). The normality of model residuals was evaluated using the Shapiro–Wilk test, and homogeneity of variances was assessed using Levene’s test.

The effects of sealer type and root region on push-out bond strength were evaluated using a linear mixed-effects model. Sealer type (Adseal, BioRoot RCS, BioRoot RCS + 5% HBN, and BioRoot RCS + 20% HBN), root region (coronal, middle, and apical), and their interaction were included as fixed effects. Tooth identity was included as a random intercept to account for the correlation among the three measurements obtained from each tooth.

Pairwise comparisons among sealer groups within each root region were performed using Tukey-adjusted estimated marginal mean comparisons. Pairwise comparisons among root regions within each sealer group were performed using Bonferroni-adjusted contrasts.

Model assumptions were assessed by examining the distribution of residuals and the homogeneity of variances. All statistical tests were two-sided, and the level of statistical significance was set at *p* < 0.05.

## 3. Results

The mean bond strength of root canal sealers, both with and without HBN, are summarized in Table 2.

SEM analysis at 2000× and 5000× magnifications revealed the structural characteristics of the RBS (Figure 1) and BCS, as well as the structural changes in the BCS following the incorporation of HBN (Figure 2). SEM analysis revealed a non-uniform distribution of HBN nanoparticles within the BioRoot RCS matrix. Larger agglomerates were observed in the BCS + 20% HBN group, whereas smaller and more evenly distributed agglomerates were identified in the BCS + 5% HBN group (Figure 2).

The linear mixed-effects analysis revealed significant effects of sealer type (*p* < 0.001), root canal region (*p* = 0.006), and their interaction (*p* < 0.001) on push-out bond strength, indicating that the differences among sealers varied according to the root canal region evaluated.

Regarding regional differences, the RBS group demonstrated significantly higher bond strength in the coronal region than in the apical region (*p* < 0.05), whereas the other regional comparisons were not statistically significant (*p* > 0.05). No significant differences among root regions were observed in the unmodified BCS group (*p* > 0.05). In the BCS + 5% HBN group, the coronal region exhibited significantly higher bond strength than the middle and apical regions (*p* < 0.05), with no significant difference between the latter two regions (*p* > 0.05). In the BCS + 20% HBN group, significant differences were observed among all three root regions (*p* < 0.05), with bond strength progressively decreasing from the coronal to the apical region.

In the coronal region, the BCS + 20% HBN group exhibited the highest mean bond strength, followed by RBS, BCS + 5% HBN, and unmodified BCS. All pairwise differences were statistically significant (*p* < 0.05).

In the middle region, RBS demonstrated the highest mean bond strength, followed by BCS + 20% HBN, BCS + 5% HBN, and unmodified BCS. No statistically significant difference was observed between RBS and BCS + 20% HBN (*p* > 0.05), whereas the remaining pairwise comparisons were significant (*p* < 0.05).

In the apical region, RBS exhibited significantly higher bond strength than unmodified BCS and both HBN-modified BCS groups (*p* < 0.05). Unmodified BCS demonstrated significantly lower bond strength than both HBN-modified groups (*p* < 0.05), whereas no significant difference was observed between the BCS + 5% HBN and BCS + 20% HBN groups (*p* > 0.05).

## 4. Discussion

Three-dimensional obturation of the root canal space and the selection of an appropriate obturation material and sealer play a crucial role in the long-term success of non-surgical root canal treatments [19]. Increasing the bond strength between dentin and obturation materials enhances resistance to root fracture and reduces the risk of microleakage, thereby contributing to the long-term preservation of endodontically treated teeth [20].

Although BCSs exhibit bioactive properties and promote hydroxyapatite formation at the sealer–dentin interface, several studies have reported lower push-out bond strength values for these materials compared with epoxy RBSs [21,22,23]. Bonding at the sealer–dentin interface is believed to occur through the chemical precipitation of calcium phosphate compounds in combination with micromechanical interlocking [24]. However, setting expansion and limited penetration into dentinal tubules may contribute to the relatively lower early bond strength observed in bioceramic-based materials [25]. Despite their relatively lower initial bond strength, BCSs are known to undergo ongoing hydration and biomineralization processes after setting, which may contribute to improved interfacial adaptation and bonding over time [26].

To improve the physicochemical properties of BCSs, the incorporation of nanomaterials has recently attracted considerable interest. Previous studies have demonstrated that nanomaterial reinforcement may improve the structural and mechanical properties of calcium silicate-based materials, suggesting their potential use as additives in endodontic sealers [11,12,27].

Therefore the present study was designed to investigate the effect of incorporating different concentrations of HBN nanoparticles into a BCS on its push-out bond strength in the coronal, middle, and apical regions of the root canal. The null hypothesis was that the incorporation of 5% and 20% HBN nanoparticles into the BCS would not significantly affect its bond strength.

According to the results of the present study, incorporation of 20% HBN significantly increased the push-out bond strength of the BCS in all three root regions. However, its performance relative to RBS varied according to root region. Therefore, the null hypothesis was rejected. In addition, a significant interaction effect between sealer type and root canal region was observed, indicating that the influence of sealer composition on bond strength varied according to the root canal level evaluated.

BCSs are generally classified into two major categories according to their chemical composition: calcium silicate-based and calcium phosphate-based sealers. BioRoot RCS is a tricalcium silicate-based root canal sealer that exhibits properties similar to mineral trioxide aggregate while overcoming several of its limitations [28]. Adseal is an epoxy RBS material containing calcium phosphate that exhibits comparable push-out resistance to AH Plus [29]. Therefore, AH Plus was considered an appropriate reference material for interpreting the bond strength performance of Adseal in the present study.

HBN is a nanomaterial that has attracted considerable interest because of its excellent oxidation resistance, mechanical strength, and chemical stability, making it a promising candidate for biomedical applications [30]. These nanoparticles possess a high surface-area-to-volume ratio, enabling substantial functional effects even at relatively low concentrations [31]. The incorporation of nanoparticles into root canal sealers is a relatively recent approach, and the available evidence remains limited, making direct comparison with previous studies difficult.

Consequently, the incorporation of inorganic nanomaterials into calcium silicate-based materials has been investigated as a means to enhance their physicochemical and mechanical properties. Viapiana et al. [12] evaluated experimental endodontic sealers based on Portland cement modified with zirconium oxide and niobium oxide, reporting variations in their physicochemical and mechanical properties. Similarly, Baghdadi et al. [10] explored the incorporation of MWCNTs, TC, and BN into BioRoot RCS, demonstrating that the effects of these additives can depend on the type and concentration of the nanomaterial used.

In a subsequent study, Baghdadi et al. [32] examined the microstructure and compressive strength of BioRoot RCS modified with MWCNTs, TC, and BN at weight concentrations of 1% and 2% in greater detail. Their findings indicated that the inclusion of 1 wt% MWCNTs and TC significantly increased compressive strength, whereas BN did not yield any notable improvement at either concentration. Furthermore, SEM observations revealed differences in particle distribution and agglomeration among the materials tested. While greater agglomeration was observed at higher concentrations of certain additives, no distinct interfacial bond was detected between the BN and the BioRoot RCS matrix. These findings indicate that the effects of inorganic fillers are not solely dependent on their intrinsic properties or concentrations; their distribution and interaction with the cement matrix can also influence the resulting material properties. Nevertheless, compressive strength reflects the overall mechanical behavior of a material and should not be considered equivalent to push-out bond strength, which evaluates resistance to displacement at the sealer–dentin interface.

In the present study, HBN powder consisting of fine particles was incorporated into the powder component of the BCS and mixed with a standardized amount of liquid without additional modification of the liquid phase. Although the present study used HBN nanoparticles rather than BN nanotubes, previous studies have demonstrated that BN nanoparticles can be incorporated into calcium-containing biomaterials and influence their mechanical properties [33]. These findings suggest that BN nanoparticles may act as reinforcing agents within calcium-based matrices. Additionally, it has been reported that nanoparticles can become separated by the cement grains [34]. Considering that dentinal tubule diameters generally range from approximately 0.5 to 3.0 µm (500–3000 nm) [35], the small particle size of HBN nanoparticles may contribute to improved adaptation of the sealer to the dentin surface and potentially enhance micromechanical interactions at the sealer–dentin interface. Another possible explanation for the present findings may be related to the physicochemical properties of HBN nanoparticles. BN has been reported to be weakly hydrophobic, exhibiting a contact angle of approximately 86° [36]. Although water absorption and solubility were not evaluated in the present study, previous research has investigated the effects of BN incorporation on the physicochemical properties of BioRoot RCS, including solubility and elution [10]. However, no statistically significant reduction in these parameters was demonstrated for the BN-modified groups compared with the unmodified material. Nevertheless, the incorporation of BN nanoparticles may potentially influence the interaction of calcium silicate-based sealers with aqueous environments. This possibility remains speculative in the context of the present study and warrants further investigation through direct assessment of water absorption and solubility.

Another finding of the present study was that the RBS showed higher push-out bond strength than the unmodified BCS in all root canal regions. Consistent with this finding, a meta-analysis reported that epoxy RBSs (AH Plus) were associated with higher push-out bond strength values than calcium silicate-based root canal sealers, irrespective of the root canal third evaluated [22]. Likewise, another study demonstrated that an epoxy RBS (AH Plus) exhibited greater dislodgement resistance than calcium silicate-based root canal sealers when the single-cone obturation technique was used [37]. The greater bond strength was attributed to the chemical adhesion of epoxy RBSs to the root structure and the covalent bonds between the exposed epoxy groups and the exposed amino groups in the collagen network of dentin [38]. Additionally, the superior adhesion of epoxy RBSs has been attributed to their ability to penetrate dentinal tubules and form covalent bonds with collagen fibrils within the dentin matrix [39]. In BCSs, tag-like structures are formed by micromechanical interaction between the root canal wall and the calcium silicate-based sealers. However, this chemical interaction, which occurs through the “mineral infiltration zone”, has been reported to create a weaker bond with dentin compared to epoxy RBSs [40]. Additionally, the dissociation into OH^−^ and Ca^2+^ ions may have increased the solubility of these materials [8] and reduced their adhesive properties. The findings reported in the literature may vary due to differences in the composition and presentation of calcium silicate-based sealers (powder–liquid/premixed) [22], as well as to methodological variations, particularly whether the materials were used alone or in conjunction with gutta-percha [37].

The incorporation of 20% HBN into BioRoot RCS may have potential clinical relevance, as it significantly improved push-out bond strength compared with unmodified BioRoot RCS in all three root regions. Nevertheless, its performance relative to the epoxy resin-based sealer was region-dependent. The BCS + 20% HBN group exhibited significantly higher bond strength than RBS in the coronal region, comparable values in the middle region, and significantly lower values in the apical region (Table 2).

These findings suggest that HBN incorporation may improve the resistance of calcium silicate-based sealers to dislodgement. Previous studies have reported differences in dislodgement resistance between calcium silicate-based and epoxy resin-based sealers [22]. However, improved push-out bond strength does not necessarily indicate superior clinical performance. Although calcium silicate-based sealers offer potential advantages related to their bioactive characteristics [24,25], the effects of HBN incorporation on setting behavior, solubility, dimensional stability, sealing ability, biocompatibility, and long-term durability remain uncertain. Therefore, the 20% HBN-modified formulation should be regarded as a promising experimental material rather than an established clinical alternative to epoxy resin-based sealers.

Another important finding of the present study was that the influence of root canal region on push-out bond strength varied among the tested sealers. A significant progressive reduction in bond strength from the coronal to the apical region was observed in the BCS + 20% HBN group, whereas the other groups exhibited different patterns of regional variation. These findings may be related to differences in smear layer removal and dentinal tubule characteristics throughout the root canal. Previous studies have demonstrated that effective smear layer removal and dentinal tubule opening can improve sealer penetration and retention [41]. However, the delivery of irrigation solutions to the apical region is often less effective, resulting in greater smear layer retention. Furthermore, the lower density and smaller diameter of dentinal tubules in the apical region may further restrict sealer penetration, thereby reducing bond strength values. In contrast, Cimpean et al. [42] reported significantly higher push-out bond strength values in the apical and middle thirds than in the coronal third for BCSs. The authors attributed these findings to the more intimate adaptation of the gutta-percha cone in the apical region. This discrepancy may be related to differences in the experimental protocols. In their study, size 40/0.08 gutta-percha cones and a 1-month storage period were used, whereas size 40/0.06 cones and a 2-week aging period were employed in the present study. It has been reported that calcium silicate-based sealers continue to experience hydration and ion exchange reactions after placement, resulting in ongoing maturation and improved interfacial bonding over time [43]. Siboni et al. stated that hydration processes in calcium silicate-based materials continue after the final setting time, allowing calcium phosphate nucleation for up to 1 month [8]. Therefore, the longer storage period used in the previous study may have enhanced the bond strength of the sealer, particularly in the apical region. In addition, differences in gutta-percha taper may have influenced the thickness and distribution of the sealer layer within the canal, which could also have contributed to the variation in push-out bond strength results between the studies.

Push-out testing is widely accepted for evaluating the dislodgement resistance of endodontic sealers. Nevertheless, the results may be influenced by several methodological variables, including obturation technique, specimen thickness, and core material characteristics [44]. Despite these limitations, the method remains a reliable approach for assessing the adhesion of root canal sealers to dentin [29,38].

In this study, SEM observations revealed a less homogeneous HBN distribution and larger agglomerates in the BCS + 20% HBN group compared with the BCS + 5% HBN group. The potential influence of this finding may be considered in light of previous studies on nanoparticle-reinforced nanocomposites, which have demonstrated that nanoparticle agglomeration can reduce the effective specific surface area and interfacial interaction between the filler particles and the surrounding matrix, potentially compromising the mechanical properties of the composite material [45,46]. Although these findings cannot be directly extrapolated to the BioRoot RCS matrix, they suggest that the larger HBN agglomerates observed at the higher concentration may have influenced the consistency of the reinforcing effect across root regions.

In addition, anatomical and structural differences along the root canal may contribute to regional variations in sealer–dentin interaction. A systematic review by Ashkar et al. [47] reported that the dentinal tubule penetration of calcium silicate-based sealers generally decreases from the coronal to the apical region. This pattern has been associated with the lower number and diameter of dentinal tubules, increased dentin sclerosis, anatomical variations, and limitations in apical irrigation. Such factors may partly explain the regional differences observed in the present study, particularly the progressive decrease in bond strength from the coronal to the apical region in the BCS + 20% HBN group. Although the bonded interface area was accounted for in the push-out bond strength calculations, differences in canal geometry and sealer layer thickness may still influence the measured values. However, since HBN distribution and agglomerate size were evaluated qualitatively, a direct causal relationship between particle agglomeration and bond strength cannot be established. Further quantitative investigations are warranted to clarify this potential relationship.

Several limitations of the present study should be acknowledged. In particular, only teeth with straight root canals were included, which may not fully represent the clinical complexity of curved root canal systems. In curved root canals, sealer penetration into the apical region may be more challenging, potentially resulting in a greater number of voids and different bond strength characteristics. Therefore, the findings obtained from curved canals may differ from those reported in the present study. In addition, only push-out bond strength was evaluated in the present study. Therefore, the influence of HBN incorporation on other important physicochemical and biological properties, including flowability, setting characteristics, solubility, dimensional stability, and biocompatibility, remains to be investigated. Another limitation of the present study is the relatively short storage period of 14 days, which does not allow for assessment of the long-term durability of HBN-modified BioRoot RCS. Further studies incorporating extended aging periods and clinically relevant aging protocols are needed to evaluate the long-term stability of its bonding performance.

In addition to storage duration, environmental conditions may influence the bonding performance of endodontic sealers. Previous studies have demonstrated that different setting environments can affect the surface characteristics and microhardness of calcium silicate-based sealers [17]. Furthermore, the influence of heat exposure on push-out bond strength appears to depend on sealer composition [48], while exposure to acidic or alkaline conditions may adversely affect the bonding performance of endodontic materials [49].

Although standardized storage conditions were used in the present study, these conditions do not fully reproduce the environmental variations that may occur clinically. Therefore, further investigations incorporating different pH levels, temperature variations, thermocycling, and prolonged aging protocols are warranted to determine the long-term bonding stability of HBN-modified BioRoot RCS.

Future studies should evaluate the behavior of HBN-modified BCSs in combination with different irrigation activation systems and laser-assisted treatment modalities, as well as compare their performance with a broader range of commercially available sealers.

Although the present findings are based on an in vitro experimental model, they provide valuable information for the preclinical evaluation of novel root canal sealers. Further studies are needed to clarify the influence of nanoparticle incorporation on the physicochemical, mechanical, and biological behavior of calcium silicate-based sealers. Additionally, clinical studies assessing the performance of nanomaterial-enriched root canal sealers are needed to validate these findings under clinical conditions.

## 5. Conclusions

The incorporation of 20% HBN nanoparticles significantly enhanced the push-out bond strength of the calcium silicate-based sealer to dentin. Within the limitations of this in vitro study, HBN incorporation appears to be a promising approach for improving the adhesive performance of BCSs. Further studies evaluating the physicochemical, biological, and long-term properties of HBN-modified sealers are required.

## Figures and Tables

**Figure 1:**
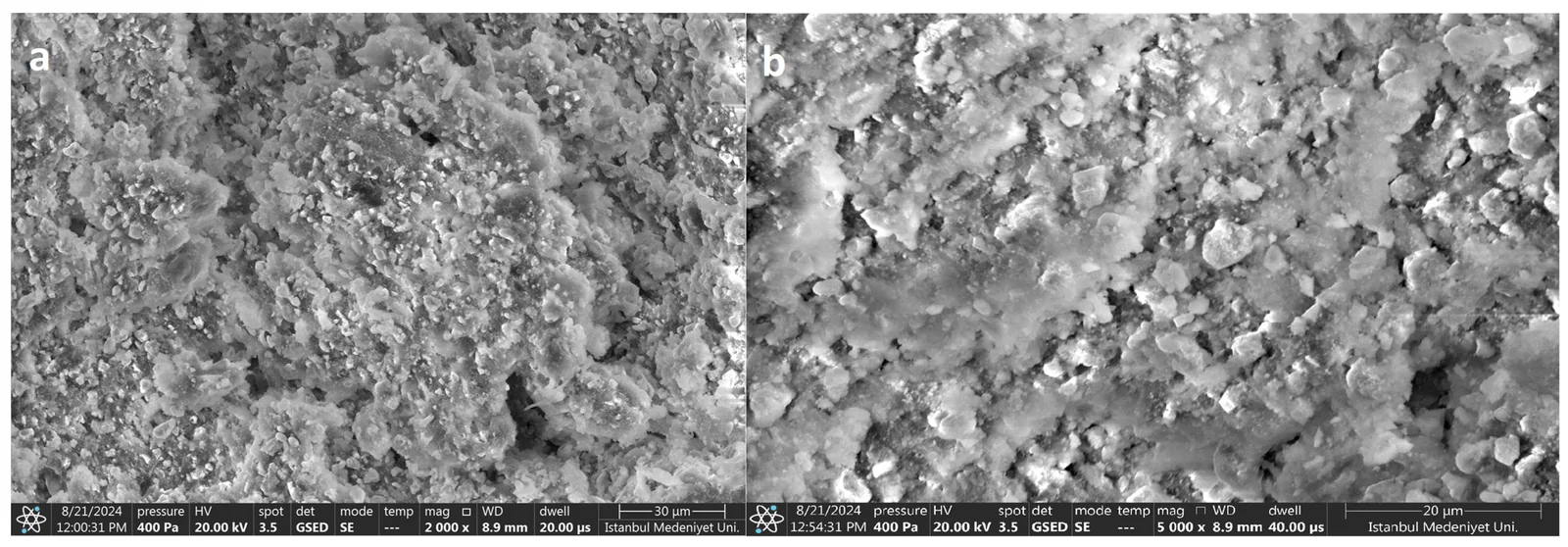
SEM micrographs showing the surface structure of the RBS at different magnifications: (**a**) 2000×, (**b**) 5000×.

**Figure 2:**
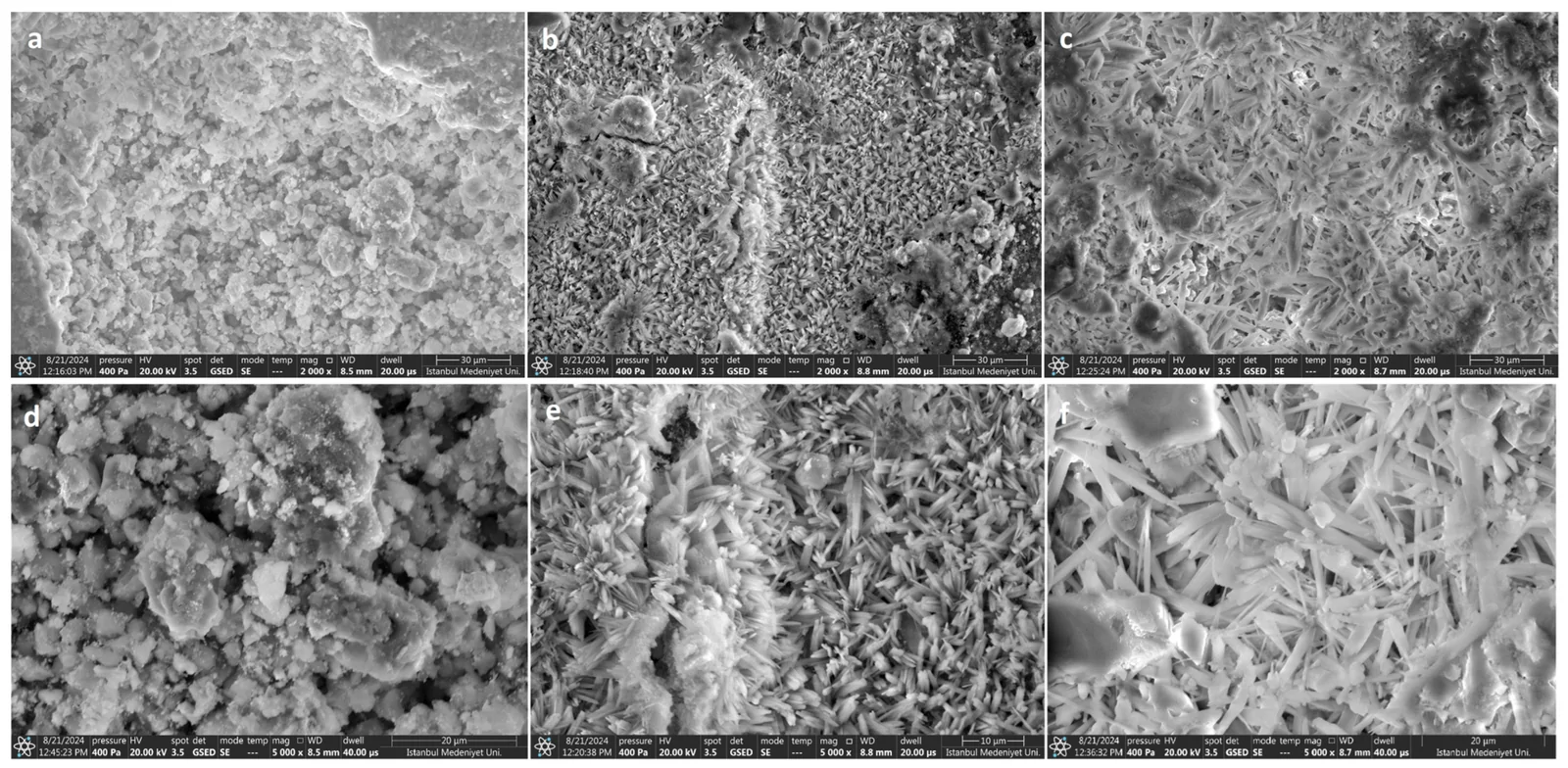
SEM micrographs showing the surface structure of BCS at different HBN concentrations and magnifications: (**a**) unmodified BCS, 2000×; (**b**) BCS with 5% HBN, 2000×; (**c**) BCS with 20% HBN, 2000×; (**d**) unmodified BCS, 5000×; (**e**) BCS with 5% HBN, 5000×; (**f**) BCS with 20% HBN, 5000×.

**Table 1:** Manufacturer and composition of root canal sealers used in this study.

Root Canal Sealers	Manufacturer	Composition	Batch/Lot Number
Adseal	Meta Biomed, Cheongju, Republic of Korea	Base: Epoxy oligomer resin Ethylene glycol salicylate Calcium phosphate Bismuth subcarbonate Zirconium oxide	ADS2509261
		Catalyst: Polyaminobenzoate Triethanolamine Calcium phosphate Bismuth subcarbonate Zirconium oxide Calcium oxide	
BioRoot RCS	Septodont, Saint Marcellin, France	Powder: Tricalcium silicate, Zirconium oxide, Povidone	B31424
		Solution: Calcium chloride, Polycarboxylate	

**Table 2:** Push-out bond strength of the tested root canal sealers in the coronal, middle, and apical root regions (mean ± standard deviation, MPa).

Root Canal Sealer	Coronal	Middle	Apical
RBS	9.59 ± 1.96 (a, x)	8.74 ± 1.32 (ab, x)	7.68 ± 1.83 (b, x)
BCS	2.23 ± 0.71 (a, y)	1.75 ± 0.51 (a, y)	2.34 ± 0.91 (a, y)
BCS + 5% HBN	7.36 ± 2.40 (a, z)	5.21 ± 0.98 (b, z)	5.68 ± 1.27 (b, z)
BCS + 20% HBN	11.35 ± 3.01 (a, w)	7.47 ± 2.76 (b, x)	5.81 ± 1.42 (c, z)

a, b, c; Different lowercase letters indicate statistically significant differences between regions within the same sealer group (Bonferroni). w, x, y, z; Different lowercase letters indicate statistically significant differences between sealer groups within the same region (Tukey).

## Data Availability

The datasets used and/or analyzed during the current study are available from the corresponding author upon reasonable request.

## Abbreviations

BCS	Bioceramic sealer
RBS	Resin-based sealer
HBN	Hexagonal boron nitride
MWCNTs	Multi-walled carbon nanotubes
TC	Titanium carbide
BN	Boron nitride
NaOCl	Sodium hypochlorite
EDTA	Ethylenediaminetetraacetic acid
SEM	Scanning electron microscopy
